# Air Pollution and Health – A Science-Policy Initiative

**DOI:** 10.5334/aogh.2656

**Published:** 2019-12-16

**Authors:** Academy of Science of South Africa, Brazilian Academy of Sciences, German National Academy of Sciences Leopoldina, U. S. National Academy of Medicine, U. S. National Academy of Sciences

**Affiliations:** 1 Academy of Science of South Africa, ZA; 2 Brazilian Academy of Sciences, BR; 3 German National Academy of Sciences Leopoldina, DE; 4 U. S. National Academy of Medicine, US; 5 U. S. National Academy of Sciences, US

## Abstract

Air pollution is a major, preventable and manageable threat to people’s health, well-being and the fulfillment of sustainable development. Air pollution is estimated to contribute to at least 5 million premature deaths each year across the world. No one remains unaffected by dirty air, but the adverse impacts of air pollution fall most heavily upon vulnerable populations, such as children, women, and people living in poverty — groups to whom States have special obligations under international human rights law. The National Academies of Sciences and Medicine of South Africa, Brazil, Germany and the United States of America are calling upon government leaders, business and citizens to take urgent action on reducing air pollution throughout the world — to the benefit of human health and well-being, to the benefit of the environment and as a condition towards sustainable development. Air pollution is a cross-cutting aspect of many UN Sustainable Development Goals.

Air pollution is a major, preventable and manageable threat to people’s health, well-being and the fulfillment of sustainable development. Air pollution is estimated to contribute to at least 5 million premature deaths each year across the world. No one remains unaffected by dirty air, but the adverse impacts of air pollution fall most heavily upon vulnerable populations, such as children, women, and people living in poverty — groups to whom States have special obligations under international human rights law.

Poor air quality threatens human life, population health, and the future prosperity of children. Air pollution also threatens the sustainability of the earth’s environment, as clean air is as vital to life on earth as clean water.

The scientific evidence is unequivocal: air pollution can harm health across the entire lifespan. It causes disease, disability and death, and impairs everyone’s quality of life. It damages lungs, hearts, brains, skin and other organs; it increases the risk of disease and disability, affecting virtually all systems in the human body.

The costs of air pollution to society and the economies of low- and middle-income countries are enormous. These economic losses are so significant that they can undercut sustainable development. Economic growth that accepts air pollution and ignores the public health and environmental impacts is unsustainable and unethical.

Combustion of fossil fuels and biomass is the most significant source of air pollution globally. These are also significant sources of short-lived climate pollutants such as black carbon, methane, ground-level ozone and the main sources of CO_2_ emissions. Many of the solutions to air pollution issues will also have a positive impact on climate change mitigation and can make important contributions to meeting a 1.5°C climate target.

Public and private investments in tackling air pollution are insufficient and do not match the scale of the problem. Opportunities to create synergies between air pollution control, climate change mitigation and sustainable development are many, but have not been fully realized.

Air pollution is a preventable problem. But without renewed action, air pollution exposure will continue to be a significant contributor to global mortality. Coupled with ageing, population growth and urbanization, more people will suffer and die each year.

Air pollution can be cost-effectively controlled through a combination of policies, legislation, regulation, standards and enforcement coupled with implementing new technologies and increasing social awareness. Air pollution control fosters economic growth and benefits national economies by averting disease and preventing productivity losses.

The National Academies of Sciences and Medicine of South Africa, Brazil, Germany and the United States of America are calling upon government leaders, business and citizens to take urgent action on reducing air pollution throughout the world — to the benefit of human health and well-being, to the benefit of the environment and as a condition towards sustainable development. Air pollution is a cross-cutting aspect of many UN Sustainable Development Goals.

Our five National Academies of Sciences and Medicine propose the adoption of a global compact on air pollution to make air pollution control and reduction a priority for all.

## Air Pollution Affects the Health of Everyone

Clean air is essential for life and health. Air pollution is the largest environmental cause of disease and early death in the world today. It has been associated with at least 5 million premature deaths every year. While air pollution impacts everyone, the burden of disease is highest among the poor and the powerless, minorities and the marginalized.

Air pollution affects people from the beginning until the end of life, causing a wide range of acute and chronic diseases from the earliest stages of child development to extreme old age. Particularly sensitive populations include infants in the womb, children, the elderly, and people with pre-existing chronic diseases. Almost all organs, systems and processes in the human body may be impacted: the lungs, the heart, the brain, the vascular system, the metabolism, and reproduction.

Air pollution is a major cause of pneumonia, bronchitis and asthma in infants and children. It slows the growth of the developing lungs of children and adolescents. It contributes to heart disease including cardiac arrhythmias and acute myocardial infarction, stroke, cancer, asthma, chronic obstructive pulmonary disease, diabetes, allergies, eczema, and skin ageing. There is emerging and growing evidence that air pollution contributes to dementia in adults and impacts brain development in children.

Women in low-income countries are disproportionately affected by exposure to household air pollution from the use of solid fuels (coal and biomass) for cooking, and they bear the greatest burden of pollution-related disease. Women also bear the main burden of caring for other household members suffering from air pollution-related ill health.

The risks of air pollution vary across societies, with vulnerability varying among individuals. Factors that affect individual vulnerability include age, gender, education, socioeconomic status, location and residence, fuels used for cooking and heating, and occupation. Biological factors that increase individual vulnerability include genetic susceptibility and underlying diseases, such as asthma, heart disease or diabetes.

Diseases related to air pollution cause productivity losses that can reduce gross domestic product, cause work and school absenteeism, and perpetuate existing societal inequalities. These diseases also result in health care costs that in rapidly industrializing countries can consume as much as 7% of national health budgets.

The global economic burden of disease caused by air pollution (both outdoor and indoor) across 176 countries was estimated to be USD 3.8 trillion in 2015. The health and economic benefits of action against air pollution will generally far outweigh the costs of action.

There is an ethical imperative to work together to protect everyone against the health risks of air pollution, which are sustained by the population as an unpaid adverse consequence of actions by polluters.

## Combustion of Fossil Fuels and Biomass is the Main Source of Air Pollution

The air pollutants of greatest concern for human health are airborne particulate matter. The unfiltered emissions of combustion contain significant concentrations of ultrafine, fine and large particles, including black carbon, as well as harmful gases.

Air pollution is a complex mixture of different components. Levels of fine particles (PM_2.5_ mass concentration) along with ozone serve as a robust indicator for regulatory purposes; with black carbon as a proxy for emissions from combustion.

The main sources of combustion-related air pollution are: A. stationary combustion facilities; B. household heating and cooking; C. controlled biomass burning and waste combustion; and D. mobile sources. The relative importance of these sources varies from country to country.

- Stationary sources include power plants, manufacturing facilities and mining with limited emission controls. Facilities that burn coal or other poor quality fuels or that rely on diesel-powered generators due to a lack of grid reliability are generally the worst offenders.
- Households are an important source of air pollution, especially in low-income countries that rely on biomass fuels for heating and cooking. They are also a place where people are greatly exposed.
- Controlled biomass burning sources related to agricultural waste burning and to land and forest clearance are important sources of air pollution in developing countries. Additional uncontrolled biomass burning is related to residential and other waste combustion.
- Mobile sources of air pollution include petroleum-powered cars, trucks, and buses; in both the private and public sectors. They are the main source of air pollution in cities. Old and poorly maintained vehicles that burn low-grade fuels are especially hazardous. Emissions from ships and aircraft are the major mobile sources of air pollution near ports and airports.

There are synergies between air pollution control and climate change mitigation as they share common sources and, to a large extent, solutions; while the majority of air pollutants also impact the climate. They also aggravate each other in multiple ways, e.g. greenhouse gases, such as methane, contribute to the formation of ground-level-ozone, and levels of ground-level ozone increase with rising temperatures and rising temperatures increase the frequency of wildfires; which in turn further elevate levels of particulate air pollution.

Black carbon from combustion impacts health but also regional temperatures, precipitation and extreme weather. The Arctic and glaciated regions such as the Himalayas are particularly vulnerable to melting as a result of deposited black carbon which heats the surface. Changing rain patterns from black carbon aerosol-cloud interactions can have far-reaching consequences for both ecosystems and human livelihoods, for example by disrupting monsoons, and droughts which are critical for agriculture in large parts of Asia and Africa.

## Call to Action

The five National Academies of Sciences and Medicine of South Africa, Brazil, Germany and the United States of America are issuing a call to action to government leaders, business and citizens to reduce air pollution in all countries. This call is underpinned by unequivocal scientific evidence on the health impacts of air pollution.

Many existing agreements, resolutions, conventions and initiatives already address aspects of air pollution. These include the Montreal Protocol, the United Nations Economic Commission for Europe Convention on Long-range Transboundary Air Pollution, the WHO Framework Convention on Tobacco Control, and the World Health Assembly resolution on the health impact of air pollution.

Therefore, the Academies propose adoption of a global compact on air pollution. This would ensure sustained engagement at the highest level and make air pollution control and reduction a priority for all. It would also encourage policymakers and other key partners, including the private sector, to integrate emission control and reduction into national and local planning, development processes, and business and finance strategies. For such a process to be successful, there would need to be both political leadership and partnerships including working together with existing multinational structures.

The Academies recognize that no perfect solution fits all situations in all countries. Nevertheless, urgent action is needed in the following areas:

There are many policy and technological solutions to reduce harmful products of combustion. For stationary sources this includes implementation of emission controls for industry and power plants or changing to clean fuels. For households this includes provision of access to clean household fuels. For controlled biomass burning this includes enforcement of rules to eliminate garbage burning and new agricultural techniques to reduce crop burning. For mobile sources this includes promoting and investing in sustainable mass transport and urban infrastructures.

Effective policies and technologies need to be shared. Where applicable, these strategies should urgently be put into action in countries at every level of economic development across the world. Some solutions enjoy a high degree of consensus. Where that consensus is lacking or where the policy choice depends importantly on context (given the heterogeneity in legal systems, geography, economic development stage, sources of pollution), tailoring of policies is needed, although there are universal actions that are needed in many parts of the world.

There is a need to collect the success stories in controlling air pollution from cities and countries and to extract lessons from those stories and share those lessons with countries now beginning to grapple with the issue.

Population exposure is directly related to population density, pollutant concentration and duration of exposure. In optimizing the costs and benefits of actions taken to improve air quality priority should be given to the pollution sources where population exposure can be reduced cost-effectively, and to reducing exposures to the poorest members of society, recognizing that these two metrics may at times conflict.

Sufficient monitoring of key pollution metrics, especially PM_2.5_ concentrations and population exposures, is a critical need in all countries. An additional need is for follow-on statistical analyses that can be used to assess the success of policy actions.

Co-benefits amongst policy instruments need to be identified. Priority should be given to policies that maximize synergies across multiple development goals, including climate change mitigation and food security. Energy efficiency improvements provide reductions in both CO_2_ and harmful products of combustion, as do many other strategies to mitigate climate change such as greater reliance on renewable energy and electrification of transport.

Efforts need to be made to devise strategies for the implementation of solutions. These strategies may include building institutional capacity, improving governance, and fostering mechanisms for cross-agency collaborations and enforcement. Using the tools of risk assessment and cost-benefit analysis will help in choosing policy designs and targets. Air pollution control policies should be designed to deliver cost-effective reductions in exposures. Ideally, they should also deliver benefits in other areas, such as climate, or other sectors, such as agriculture. Polluters could be incentivized to find the cheapest ways of reducing pollution and thereby exposures.

This call for action requires mobilizing finance and substantial investment in opportunities to reduce air pollution. Increased funding is also needed for research, pollution monitoring, infrastructure, management and control, and stakeholder interaction.

Finally, there needs to be advocacy for action where citizens are informed and inspired to reduce their air pollution footprint and advocate for bold commitments from the public and private sectors.

## Author Information

### Academies Working Group

Maria de Fatima Andrade, Professor of Meteorology and Atmospheric Sciences, University of São Paulo, São Paulo, Brazil.

Paulo Artaxo, Professor of Environmental Physics, University of São Paulo, São Paulo, Brazil.

Simone Georges El Khouri Miraglia, Associate Professor and Leader of the Laboratory of Economics, Health and Environmental Pollution (LESPA), Federal University of São Paulo, São Paulo, Brazil.

Nelson Gouveia, Associate Professor of Epidemiology, University of São Paulo, São Paulo, Brazil.

Alan J. Krupnick, Senior Fellow, Resources for the Future, Washington, DC, U.S.A.

Jean Krutmann, Scientific Director, IUF — Leibniz Research Institute for Environmental Medicine, Düsseldorf, Germany.

Philip J. Landrigan, Professor of Biology and Director, Program in Global Public Health and the Common Good, Boston College, Boston, U.S.A.

Kristy Langerman, Senior Lecturer, University of Johannesburg, Johannesburg, South Africa.

Tafadzwa Makonese, Senior Researcher and Lab Manager, University of Johannesburg, Johannesburg, South Africa.

Angela Mathee, Director MRC Environment & Health Research Unit, South African Medical Research Council (SAMRC), Johannesburg, South Africa.

Stuart Piketh, Professor of Environmental Science, North-West University, Potchefstroom, South Africa.

Beate Ritz, Professor of Epidemiology and Environmental Health Sciences, University of California, Los Angeles, U.S.A.

Paulo H. N. Saldiva, Director, Institute of Advanced Studies, University of São Paulo, São Paulo, Brazil.

Jonathan Samet, Dean, Colorado School of Public Health, Aurora, U.S.A.

Tamara Schikowski, Head of Research Group “Environmental epidemiology of lung, brain and skin aging”, IUF — Leibniz Research Institute for Environmental Medicine, Düsseldorf, Germany.

Alexandra Schneider, Head of Research Group “Environmental Risks”, Institute of Epidemiology, Helmholtz Zentrum München — German Research Center for Environmental Health, Neuherberg, Germany.

Kirk R. Smith, Professor of Global Environmental Health, University of California, Berkeley, U.S.A. and Director, Collaborative Clean Air Policy Centre, Delhi, India.

Claudia Traidl-Hoffmann, Chair and Institute of Environmental Medicine, UNIKA-T, Technical University of Munich and Helmholtz Zentrum München — German Research Center for Environmental Health, Augsburg, Germany.

Alfred Wiedensohler, Head of Department for Experimental Aerosol and Cloud Microphysics, Leibniz Institute for Tropospheric Research, Leipzig, Germany.

Caradee Wright, Specialist Scientist, South African Medical Research Council (SAMRC), Parktown, South Africa.

### Invited External Experts

David Richard Boyd, United Nations Special Rapporteur on Human Rights and the Environment, Office of the United Nations High Commissioner for Human Rights (OHCHR), Geneva, Switzerland.

Valentin Foltescu, Senior Science and Programme Officer, Climate and Clean Air Coalition Secretariat, United Nations Environment, New Delhi, India.

Richard Fuller, Lancet Commission on Pollution and Health Co-Chair, Pure Earth and Global Alliance on Health and Pollution, New York, U.S.A.

Dorota Jarosińska, Programme Manager, World Health Organization, European Centre for Environment and Health, Bonn, Germany.

Jacqueline Myriam McGlade Former Chief Scientist, United Nations Environment, Nairobi, Kenya.

Drew Shindell, Duke University Durham, NC, U.S.A. and Chair of the Scientific Advisory Panel, Climate and Clean Air Coalition, Paris, France.

### Secretariat

Marcos Cortesao Barnsley Scheuenstuhl, Executive Director of International Affairs, Brazilian Academy of Sciences (ABC), Rio de Janeiro, Brazil.

John P. Boright, Director of International Affairs, U.S. National Academy of Sciences (NAS), Washington, DC, U.S.A.

Siyavuya Bulani, Senior Liaison Officer, Academy of Science of South Africa (ASSAf), Pretoria, South Africa.

Margaret Hamburg, Foreign Secretary, U.S. National Academy of Medicine (NAM), Washington, DC, U.S.A.

Kathrin Happe, Deputy Head of Department of Science — Policy — Society, German National Academy of Sciences Leopoldina, Halle (Saale), Germany.

Jan Nissen, Senior Officer, Department of International Relations, German National Academy of Sciences Leopoldina, Halle (Saale), Germany.

Isabel Scheer, Assistant, Department of International Relations, German National Academy of Sciences Leopoldina, Halle (Saale), Germany.
